# Researching teaching-learning concepts in the health professions using document analyses?

**DOI:** 10.3205/zma001801

**Published:** 2026-01-15

**Authors:** Jan-Hendrik Ortloff, Manfred Fiedler, Nils Boelmann, Daniela Schmitz

**Affiliations:** 1 Witten/Herdecke University, Department of Human Medicine, Witten, Germany

**Keywords:** document analysis, website analysis, higher education research, evaluation strategy

## Abstract

This article explores the question of how document analyses can be used as a research methodology in academic training resp. teaching and learning research. Documents are texts of different origins, types and quality that are not influenced by researchers, were not created for the research itself and can therefore be understood as objectifications of social reality.

Therefore, they contain research-relevant information for which certain conditions of origin are unknown at the same time. In academic teaching, documents such as examination regulations often standardise or structure the practice of teaching and learning and are therefore particularly relevant for academic training and teaching-learning research.

The decisive factor for the use of document analysis is the research interest. Documents are often used as a first or additional data source. Document analyses are then usually part of a multi-method approach or are used in preparation for further research steps or to examine relevant research results with regard to their significance. The increase in non-textual, multimodal documents means that document analyses are becoming increasingly important as an independent method in research. The article reflects on the method using a case study of a two-stage document analysis in the form of a website analysis and a textual document analysis.

## Abstract (de)

In diesem Beitrag wird der Frage nachgegangen, wie Dokumentenanalysen in der akademischen Ausbildungs- bzw. Lehr- und Lernforschung als Forschungsmethodik eingesetzt werden können. Dokumente sind von Forschenden unbeeinflusste Texte unterschiedlicher Herkunft, Art und Güte, die nicht für die Forschung selbst geschaffen wurden und dadurch als Objektivationen sozialer Wirklichkeit verstanden werden können.

Daher beinhalten sie forschungsrelevante Informationen, bei denen gleichzeitig bestimmte Entstehungsbedingungen unbekannt sind. In der akademischen Lehre sind Dokumente, wie etwa Prüfungsordnungen, die Praxis des Lehrens und Lernens häufig normierende oder strukturierende Texte und haben daher besondere Relevanz für akademische Ausbildungs- bzw. Lehr-Lernforschung.

Entscheidend für den Einsatz der Dokumentenanalyse ist das forschungsleitende Interesse. Dokumente werden dabei häufig als erste oder zusätzliche Datenquelle genutzt. Dokumentenanalysen sind dann meist Bestandteil eines Multimethodenansatzes bzw. werden vorbereitend auf weitere Forschungsschritte oder prüfend in Hinsicht darauf relevante Forschungsergebnisse in Hinsicht auf ihre Bedeutung eingesetzt. Die Zunahme nicht rein textlicher, multimodaler Dokumente lässt die Dokumentenanalyse als eigenständige Methode in der Forschung an Bedeutung gewinnen. Der Beitrag reflektiert die Methode anhand eines Fallbeispiels einer zweitstufigen Dokumentanalyse in Form einer Webseitenanalyse und einer textlichen Dokumentenanalyse.

## 1. Document analysis as a research method

Documents such as expert standards or guidelines are of particular importance for healthcare, as they are intended to provide orientation for healthcare practice. The same applies to university documents, such as curricula or study and examination regulations, which influence or even standardise the practice of teaching and learning in the studies of future professionals in various ways.

In contrast to most qualitative and quantitative data collection methods, document analyses are based on the fact that documents already exist before the research project and have been created as carriers of information for their own purpose, uninfluenced and uninfluenceable by the researcher and the research (non-reactive) [1]. This functionality of documents distinguishes them on the one hand from research-generated texts or multimodal data, and on the other hand from scientific texts created by researchers themselves, whose creation directly serves the “publication of knowledge” [2]. 

There is often a narrow understanding of documents in written-textual format, such as personal or ego documents (e.g. diaries), internal or official documents of organisations [3]. In a broad understanding, however, documents are multifaceted and offer multimodal access points for research: “They are understood as data or carrier substances of content; they are to be read critically as sources; we encounter them as traces with an indirect referential character, as media with a mediating function, as ambiguous signs or as adversaries with their own logic – they are thereby also generally named as things, objects, items, stuff, materials, containers, props, utensils, artefacts, testimonies, circumstantial evidence, symbols, nomads, witnesses, guarantors, agents, actants, actors, etc.” ([4], author’s translation). In this respect, documents can have a verbal-textual, visual, audiovisual, multimedia or hypermedia character. 

In principle, documents are characterised by the fact that they are objectifications of social practices in their respective form [4]. In the context of education and higher education research, they can therefore express the intentions of educational programmes and the regulations governing the design of teaching and learning programmes. When analysing documents, it is therefore important to consider the context in which they were created and used as a social context. The subject of the analysis can be the content or the function or the context of use, but also the context in which the documents were created.

**Figure 1:**
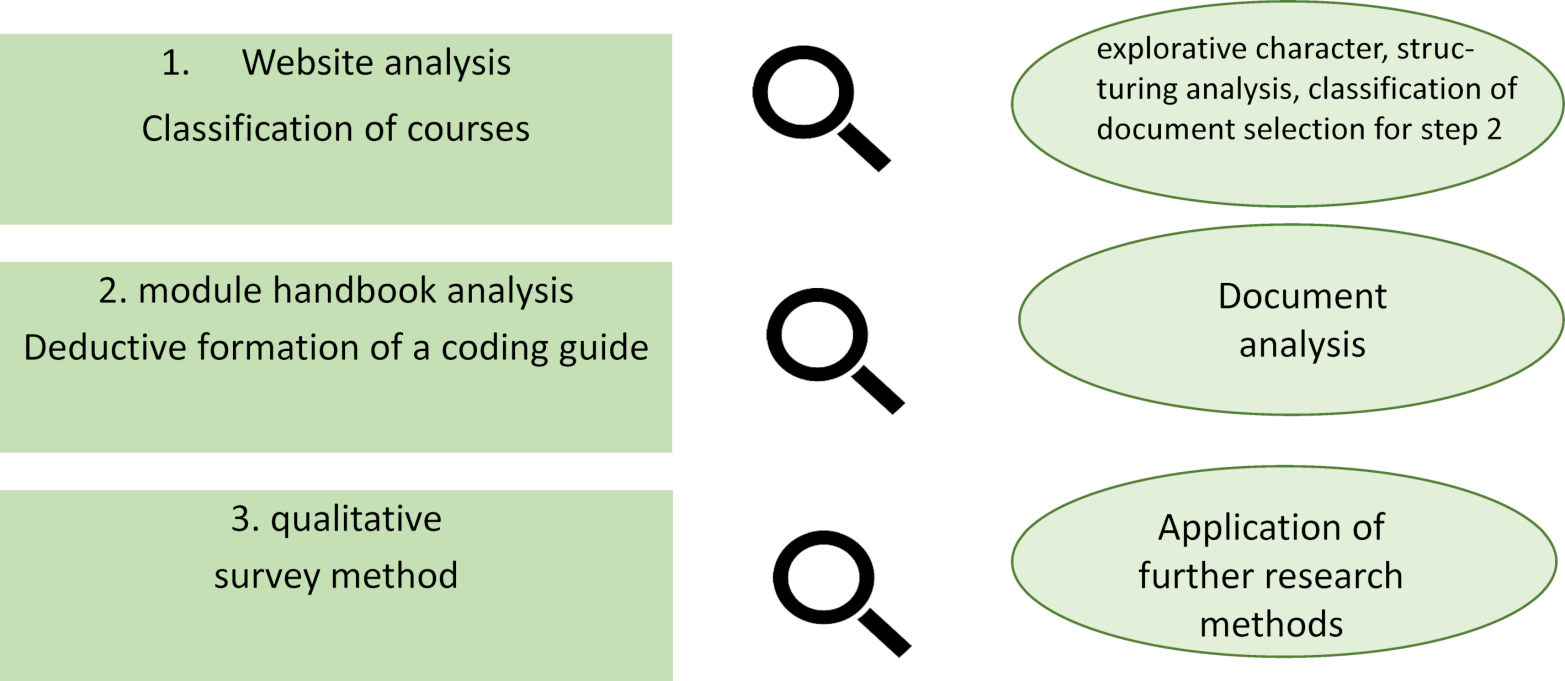
Document analysis procedure

The understanding of document analyses receives comparatively little attention in the scientific-methodological discussion [5]. This is also surprising because, for example, websites as digital documents in the context of big data [6] have been part of scientific and commercial practice for years with regard to quantitative analyses, especially in social media research. Document analyses are also not fundamentally alien to teaching and learning research in the health sciences, but are only reflected to a limited extent in the discussion of methods in research. In the following, we will discuss the benefits, but also the limitations of document analysis and exemplify them using a current research project to analyse teaching-learning concepts (see figure 1 (Fig. 1)).

## 2. Document analyses and higher education research

### 2.1. Preconditions for document analysis

The strengths of document analyses include research-economic aspects of data collection when it comes to easily accessible documents, often in large quantities via the Internet, which can be analysed from the desk [7]. In addition, documents allow direct insights into the practices documented therein and in some cases conclusions about social conditions or circumstances [4]. There is often also a variety of documents that are available and do not have to be produced as part of the research.

A disadvantage of the non-reactivity of documents is the lack of insight into the contextual conditions, e.g. the authorship of online documents or the use of the documents by the addressees [7]. The accessibility of documents can also be limited or even non-existent. A further disadvantage lies in the informative value of the documents, which were produced for a specific purpose that does not fully coincide with the research problem. Based on the current state of research, some authors also reflect on the use of the document analysis method in their studies and come to the conclusion that limitations lie in access to the documents, in their topicality and informative value. The latter aspect in particular is reflected in the fact that there can be a discrepancy between the content listed in module handbooks and the reality of teaching and learning [8], [9].

### 2.2. Document analysis and training research

Nowadays, document analyses are used in many different ways as a research method. In the context of educational research in healthcare professions, Bauer et al. [10] used document analyses to analyse study and examination regulations and freely accessible module handbooks in human medicine in order to determine the scope and integration of learning content for scientific work in medical degree programmes. Trojan et al. [11] analyse module handbooks with regard to learning content on evidence-based research in bachelor’s and master’s degree programmes in the field of public health. Erdwiens et al. [12] analysed module handbooks for social work degree programmes to determine the extent to which content on the topic of digitalisation is provided.

Module handbooks as documents are ultimately to be understood as module descriptions that contain information on content, qualification objectives, forms of teaching and learning, participation requirements and workload [8] and thus come close to academic reality [12]. However, Hohenstein et al. [9] point out that there are major differences in the design of curricula and that the degree of concretisation of module handbooks can vary. Summing up the state of research on the use of document analyses, we note that these are often carried out in preparation for the use of other methods, such as qualitative interviews. The document analysis is then an explorative preliminary stage for further surveys.

## 3. Principles of data collection and evaluation in document analyses

Documents are texts that are uninfluenced by the researcher and therefore have intrinsic validity [13], which is why they are often referred to as natural data [3]. It may be in the research interest either to typify and analyse them with regard to their document characteristics, their morphological features, or to use them as a carrier of information, as a data source to answer a research question and thus primarily for content analysis [1].

In both cases, the selection of the documents to be analysed is a crucial research task. In the classical sense [14], the aim is to develop inclusion criteria that make it possible to achieve a theoretically saturated data basis in terms of answering the research question. This is not least due to the fact that documents are always purposefully integrated and cannot be understood independently of context. They are an expression of a social practice, have a history and an environment of creation and further development [15], which determines the form, structure and content of a document.

### 3.1. Theoretical framework

Döring and Bortz [7] attribute a qualitative character to documents, which can be analysed using interpretative-qualitative data analyses. As in other qualitative data collection methods, reference or the development of a theoretical framework is fundamental for document analyses. Inductive methods, such as open coding, are only suitable for document analyses to a limited extent, as there is a particular risk of subjectivising results due to the lack of reference to the research questions when the documents are created. Depending on the type of document, e.g. a website, the data material and the object of research are distributed unspecifically in the document with regard to the research interest. The theoretical framework structures the methodological procedure in document analysis in particular, as it enables the definition of a coding guideline with which the document can be systematically analysed.

Theoretical concepts can be derived from the specific context of the documents. In this context, Asdal and Reinertsen speak of “document ethnography” [15]. As a rule, however, they are also derived in document analysis primarily from the research question and thus from a deductive pre-understanding of the research field.

### 3.2. Content analysis

In German-speaking countries, Mayring [14], among others, has drawn up a four-step investigation plan for document analyses as part of his qualitative content analysis. In the first step, a research question is developed, for which the material to be analysed is determined in the second step. In the third step, researchers must check the quality and appropriateness of the documents to be analysed. Mayring lists six criteria for carrying out a so-called source criticism: Type and origin of the documents, internal and external characteristics, intentionality of the documents and proximity to the subject matter.

The READ approach by Dalglish et al. [16] is a more concrete procedure. READ stands for

1. Ready your materials,
2. Extract data,
3. Analyse data and
4. Distil your findings.

In the first step, the type and quantity of documents to be included is determined according to the research question and their accessibility is checked. Depending on the research question and the type of documents, the procedure for the data to be extracted is determined in the second step. Dalgish et al. [16] suggest a tabular approach, for example in the case of a deductive approach, in which the documents analysed are listed horizontally and the categories to be found are entered vertically, as in a cross-tabulation. Based on the methodology of grounded theory, initial notes and ideas on theory formation should be recorded in memos and, if necessary, the procedure should be adapted, particularly with regard to category formation. In phase three, this procedure is finalised in the sense of an initial evaluation or theory formation. Questions regarding the quality and coherence of the analysed documents as well as data saturation must be clarified. The phase of summarising and structuring what has been found in the documents begins when the researchers determine that either the documents included in total can adequately represent the criteria relevant to the research, or when only very specific criteria can be used in the analysis over time, or when the researchers finally believe that they have found satisfactory answers to the research question and the phenomenon to be investigated.

### 3.3. Website analyses

Websites are often analysed with regard to certain properties that primarily concern the suitability for the supposed or intended purposes of websites. These include accessibility [17], especially for reference groups, or the appropriateness of the design, customising with regard to the use by and communication with the preferred visitors to the website (usability, user/respondent experiences), whereby these make use of website test procedures, for example, which are often supplemented by user surveys or questionnaires [18], [19].

Content analyses of websites, on the other hand, mainly refer to defined website content, i.e. parts or subdomains of websites. In addition to the classic content analysis approach, as is often used in other survey methods, alternative content analysis methods with often digital, programme-based recording and analysis tools are used, particularly for large amounts of data or complex website structures, such as websites with chat histories, social media content or blog content [20], which are usually quantitatively oriented. 

### 3.4. Quantitative analysis

Another important method is quantitative content analysis. This involves the statistical evaluation of relevant data in selected documents, such as ventilation times in intensive care units when analysing intensive care protocols. Another approach is to analyse the frequency and frequency distributions of items and codes in documents (“analysis of category frequencies” [4]), which is usually carried out using digital analysis tools. The determination of word frequencies (“frequency analysis”) can also be used to assess the relevance of certain discussions and content and can therefore also be part of inductive categorisation [21].

Application example for a document analysis: Factors influencing teaching-learning formats at private universities 

The joint project of TU Dortmund University and Witten/Herdecke University, funded by the Federal Ministry of Research, Technology and Space is investigating factors influencing teaching-learning formats at private universities (ELLpH) in three sub-projects. In the context of the sub-project referred to here, the focus lies on the conditions of introduction and the processes of implementing (innovative) teaching-learning formats. The project focuses on the fields of medicine/health and psychology, as well as management and business administration.

The multi-method project consists of two phases, an initial document analysis and a subsequent interview phase with lecturers and programme directors. The document analysis itself, which has now been completed, consisted of a two-stage process. Two different document types were analysed: websites on the one hand and module handbooks on the other. While module handbooks are classic, written texts, which today are mostly available in digital form as PDF files, a large part of which was directly available on the websites , the websites are usually multimodal documents consisting of written texts as well as auditory, visual or audio-visual elements, and which have a structured layout in the form of subdomains, such as the degree programmes analysed.

The website analysis had an exploratory character in order to gain a structured overview of the research field and to typify universities and study programmes in order to enable sufficient saturation of the data in the further course of the document analysis. This analysis therefore served to cluster the documents in the research field and thus rationalised the selection of documents for the second part of the document analysis in the form of the analysis of module handbooks.

Due to this typifying and structuring approach, the theoretical foundation of the procedure was kept simple in terms of category formation, but was strictly defined in order to avoid an interpretative approach when examining the research field and was therefore primarily descriptive in nature. This means that only a few categories were used for the analysis, but these were evaluated and assigned with regard to the tests of the characteristics without free (inductive) codes. For this purpose, analogous to Dalglish et al. [16], a cross table was created for each website analysed, in which all degree programmes of the subject disciplines investigated were recorded and the deductive categories were assigned to them. The interpretative procedures [22] for interpreting the results, which are also quite common in document analysis, played a subordinate role, as the website analysis has a preparatory character for the analysis of the module handbooks and an interpretative approach can subjectively influence the formation of categories by researchers and thus complicate the selection decision of the textual documents (module handbooks). Finally, the website analysis enables the specification of questions as objectified preliminary “findings”.

The second stage, in the form of analysing module handbooks, had the purpose of gaining primary insights into teaching-learning formats. The websites to be included were selected both according to fields of study and on the basis of the typifications formed in the website analysis, above all study programmes and types of higher education institution. The theoretical framework was significantly expanded. In particular, didactic and professional association guidelines, such as those of the German Psychological Society (Deutsche Gesellschaft für Psychologie) [23] or recommendations on academic training in the health professions by the German Council of Science (Wissenschaftsrat) [24] were used. A theoretical frame of reference was thus chosen which is assumed to be relevant both with regard to the health science subjects and with regard to the normative conditions for the implementation of teaching-learning concepts. It was noticeable that such a priori guidelines in the form of association or similar documents are widespread in the health science subjects, but are absent in the comparative field of business studies, apart from a non-binding international basic curriculum. In view of the broad theoretical framework, a differentiated coding paradigm was created and each analysed document was evaluated in its own evaluation mask. The evaluation and presentation of results were analysed in terms of content. 

The completion of the document analysis makes it possible to draw initial conclusions, for example with regard to certain types of private universities and the principles of the use of teaching-learning formats. At the same time, these interim results allow questions to be asked of the material that can be used as a basis for the interview phase of the project.

## 4. Conclusion and outlook

Document analysis is an interesting and increasingly used method for higher education research, as degree programmes and studies at universities are structured by documents such as study and examination regulations, module handbooks, curricula for teachers and students, which are supplemented by digital, website-based texts and university-specific digital media and platforms. They thus provide information on subject-specific and didactic concepts and at the same time represent types of documents that can themselves be the subject of higher education research. Multimodality, for example within digital teaching-learning formats such as serious games, digitalised scenario-based learning, VR or teaching videos, make document analysis more significant for academic teaching-learning research, as it can be used to directly investigate the characteristics of teaching-learning formats. 

It is particularly important to consider the particular strengths and limitations of document analysis in the context of a research project. Since documents are created by researchers for their own purpose without being influenced by them [25], the comparative analysis of documents in terms of type, characteristics and fulfilment of purpose is an obvious object of research. When documents are included with regard to specific research questions, they generally have a limited scope. This applies in particular to documents that, like module handbooks, process standards or guidelines, are always written in advance of actual practice. They are therefore instruments for standardising lived practice, but not yet the practice itself.

Through document analyses, researchers cannot generate any content other than that which is hidden in the documents, which is intended by the mostly unknown creators of the document [26]. This means that only parts of the document remain relevant, at least for content-related research questions, which also harbours the risk of selectively choosing documents because they are considered more productive. In contrast to this risk of bias distortion, the non-reactivity of the document [26] means that it is uninfluenced as research material, for example by the relationship between the researchers and the object of research, which therefore cannot or should not have any influence on the results.

Document analyses are not least a research instrument that enables the concretisation of research questions in practice, because documents are neither a distillate nor a comprehensive image of practice, but a part of it, however significant. Document analyses are therefore a useful research instrument, especially as preparation for further survey procedures as part of a multi-method approach. They are particularly suitable for educational research, for example, when the requirements for teaching practice can be analysed in terms of implementation and actual practice by means of standard-setting documents.

Particularly in view of the strengths and limitations of the method, the rather sparse debate in the discussion of methods is incomprehensible, as it is sometimes used with little reflection or borrowed from concepts of other research methods. This may also be due to the fact that documents are already available prior to the research project and therefore, in comparison to traditional survey instruments, little or no effort is required on the part of the researchers, albeit depending on accessibility. This advantage in terms of research economics can ultimately be better utilised if document analysis is considered and used as an independent method as part of a more in-depth reflection on methods.

## Authors’ ORCIDs

- Jan-Hendrik Ortloff: [0009-0002-1218-3097]
- Daniela Schmitz: [0000-0002-4874-0847]

## Competing interests

The authors declare that they have no competing interests.
